# Collagen regulates the ability of endothelial progenitor cells to protect hypoxic myocardium through a mechanism involving miR‐377/VE‐PTP axis

**DOI:** 10.1111/jcmm.13712

**Published:** 2018-07-25

**Authors:** Ana‐Maria Rosca, Daniel Nicolae Mitroi, Valeriu Cismasiu, Rodica Badea, Georgiana Necula‐Petrareanu, Mihai Bogdan Preda, Cristina Niculite, Raluca Tutuianu, Stefan Szedlacsek, Alexandrina Burlacu

**Affiliations:** ^1^ Institute of Cellular Biology and Pathology “Nicolae Simionescu” Bucharest Romania; ^2^ “Victor Babes” National Institute Bucharest Romania; ^3^ Institute of Biochemistry of the Romanian Academy Bucharest Romania

**Keywords:** angiogenesis, endothelial progenitor cells, in vitro cell transplant, microRNA‐377, myocardium, protein tyrosine phosphatases

## Abstract

The possibility to employ stem/progenitor cells in the cardiovascular remodelling after myocardial infarction is one of the main queries of regenerative medicine. To investigate whether endothelial progenitor cells (EPCs) participate in the restoration of hypoxia‐affected myocardium, we used a co‐culture model that allowed the intimate interaction between EPCs and myocardial slices, mimicking stem cell transplantation into the ischaemic heart. On this model, we showed that EPCs engrafted to some extent and only transiently survived into the host tissue, yet produced visible protective effects, in terms of angiogenesis and protection against apoptosis and identified miR‐377‐VE‐PTP axis as being involved in the protective effects of EPCs in hypoxic myocardium. We also showed that collagen, the main component of the myocardial scar, was important for these protective effects by preserving VE‐PTP levels, which were otherwise diminished by miR‐377. By this, a good face of the scar is revealed, which was so far perceived as having only detrimental impact on the exogenously delivered stem/progenitor cells by affecting not only the engraftment, but also the general protective effects of stem cells.

## 1 INTRODUCTION

Over the past decades, regenerative medicine has focused on the discovery and understanding of stem and progenitor cell populations for cardiovascular remodelling after myocardial infarction. It came out that both angiogenesis and neovascularization induced by stem/progenitor cells are essential processes for improving cardiac function in pathological settings associated with cardiac ischaemia.^1^, ^2^ Among the progenitor cells with cardioprotective effects, the “late‐outgrowth” endothelial progenitor cells (EPCs) have emerged as having strong proliferative potential and a high capacity to become incorporated into the newly developing vessels.^3^

These cells have been extensively used in various studies both in vitro and in animal models, and several clinical trials have been initiated to evaluate their therapeutic potential for cardiac regeneration.^4^, ^5^, ^6^ However, no reliable in vitro system able to mimic cell transplantation and predict the in vivo clinical therapeutic outcome exerted by stem/progenitor cells has been established so far.

To study the mechanisms underlying the beneficial outcome of EPC therapy on the myocardial ischaemic tissue, we established an in vitro cell transplantation model that allows in‐depth interaction between EPCs and myocardial tissue. We present here data showing that co‐culture of cardiac slices with EPCs led to reduced cardiac cell ischaemia and apoptosis, as compared to cardiac slices alone, as a consequence of the paracrine protective effects of EPCs on the hypoxic heart. Furthermore, a systematic analysis of the functional relationship between expression of protein tyrosine phosphatases (PTPs) and microRNAs revealed that during interaction between EPCs and hypoxic myocardial tissue, miR‐377‐VE‐PTP axis was involved in the angiogenic effects of EPCs in ischaemia‐affected myocardium.

## 2 MATERIALS AND METHODS

### 2.1 EPC isolation

Primary cultures of EPCs were isolated from human umbilical cord blood, as previously described.^7^ The cells were grown in EGM‐2 Bullet Kit medium (Lonza) onto collagen‐coated plates and used between the 8th and 12th passages.

### 2.2 Matrigel assay

EPCs were resuspended in EGM‐2 medium (10^5^ cells/mL) and 100 μL cell suspensions were added in each 96‐well plate well over 50 μL Matrigel. After 24 hours of incubation, the tube‐like structures were photographed under a VERT.A1 ZEISS microscope and the number of closed structures, branching points and total tube length per field were determined using ImageJ software, NIH, USA.

### 2.3 Preparation of the mouse cardiac slices

Mice were housed and used in accordance to national and EU regulations for animal experimentation (Directive 2010/63/EU of the European Parliament) and all procedures were approved by the Institutional Ethical Committee of the Institute of Cellular Biology and Pathology “Nicolae Simionescu” Bucharest. Animals were killed by cervical dislocation and the heart was immediately harvested and rapidly washed in ice‐cold Tyrode solution (137 mmol/L NaCl, 2.7 mmol/L KCl, 1 mmol/L MgCl_2,_ 1.8 mmol/L CaCl_2,_ 0.2 mmol/L Na_2_HPO_4,_ 12 mmol/L NaHCO_3,_ 5.5 mmol/L D‐glucose). The atria were removed and the ventricular myocardium was immediately embedded in 4% low melting point agarose (Invitrogen) prepared in Tyrode solution, according to a protocol described by Halbach et al.^8^ Then, the myocardium was transversely sliced into 300 μm‐thick sections, using a Leica vibratome VT1200S.^9^ The sections were kept in oxygenated cold Tyrode solution for 30 minutes before being used for co‐culture experiments.

### 2.4 Co‐culture of myocardial slices with EPCs

This was carried out in a modified one‐well culture plate (BD Falcon), based on an ICBP's patented system (Official Bulletin of Industrial Property, Section Patents, no 5/2005, page 19). Briefly, a circular incision was carried out at the bottom of the well and a glass coverslip was glued underneath to create a well of 800 μm depth (the thickness of the bottom of the culture plate in the well centre) and 5 mm in diameter. The co‐culture system was thereafter re‐sterilized individually by exposure to ultraviolet light for 30 minutes.

Myocardial slices (300‐μm) were placed on the bottom of each well in 15 μL culture medium containing EPCs. The wells were ultimately covered with a sterile round 12 mm coverslip (Marrienfield) to prevent evaporation. The optimal humidity of the co‐culture system was created by introducing 3 mL of sterile water in the groove outside the well. The co‐culture was maintained at 37°C in a 5% CO_2_ atmosphere and 98% humidity for 24 hours. Then, the cardiac slices were removed, washed in PBS and either analysed for cell adherence and engraftment or moved into 96‐well plates for further culture in 100 μL EGM‐2 complete culture medium for various times. In experiments following the engraftment of EPCs into the cardiac tissue, cells were pre‐labelled with Flash‐Red beads (660‐690 nm, 1,63 μm, Bangs Laboratories, for 24 hours, as previously reported^7^) or Vybrant‐CM‐DiI (Molecular Probes, in accordance to the manufacturer's instructions for labelling cells in suspension) before co‐culture. The engraftment was images by IVIS Spectrum CT System (Perkin Elmer, Caliper, LifeSciences) and confocal microscopy (Leica TCS‐SP5).

### 2.5 Caspase‐3 assay

To determine the effect of EPCs on the apoptosis of the cells of the hypoxic heart slices after in vitro co‐culture, Caspase‐3 activity was measured using a colorimetric assay kit (R&D Systems). Briefly, the cardiac sections were homogenized in 100 μL Lysis Buffer by employing an Eppendorf micropestle and incubated on ice for 15 minutes. The lysate obtained by centrifugation (10,000 *g*, 1 minute, 4°C) was collected and 50 μL aliquots were combined with 50 μL reaction buffer and 5 μL DEVD‐AFC substrate. The mixture was incubated at 37°C for 1 hour and the substrate cleavage was quantified spectrophotometrically with a Tecan Infinite 200 spectrofluorometer at a wavelength of 405 nm. The data were normalized to the protein concentration determined by BCA assay.

### 2.6 Quantification of EPC proliferation in co‐culture system

This determination was based on the analysis of human‐specific Alu sequences by Real‐Time PCR.^10^ Shortly, genomic DNA was extracted from EPC‐heart slice co‐cultures using High Pure PCR Template Preparation Kit (Roche). Real‐time PCR was performed from 1 μg gDNA using SensiFAST™ SYBR^®^ No‐ROX Kit (Bioline) and the LightCycler 480 System (Roche Life Science). The comparative C_T_ method was used to quantify the results, assuming a 100% reaction efficiency.

### 2.7 Determination of VEGF by ELISA assay

To quantify VEGF secreted by mouse cardiac cells in co‐culture with human EPCs, DuoSet ELISA Development System for mouse VEGF (R&D Systems) was used, with no cross‐reactivity or interference with human forms of VEGF. Briefly, the culture medium was collected at various time‐points after co‐culture and centrifuged to remove cellular debris, before being assayed by ELISA, following the manufacturer's instructions.

### 2.8 Quantification of the expression of miRNAs in EPCs

Total RNA was extracted with TRI Reagent(R) (Sigma Aldrich) from cultured EPCs and evaluated with NanoDrop ND‐1000 Spectrophotometer (Thermo Scientific). For cDNA synthesis, the QuantiMir RT Kit (System Biosciences) was used, according to manufacturer instructions. cDNA templates were quantified in a real‐time SYBR Green qPCR, with the Quant Studio 7 Flex Real‐Time PCR System (Applied Biosystems), using universal reverse primers and miRNA‐specific forward primers. U6 snRNA was used as endogenous control and for data normalization. The C_T_ for the analysed miRNAs was subtracted from those of the endogenous control.

### 2.9 Assessment of miR‐377

The expression of miR‐377 in EPCs was altered by transfection with Stemfect™ RNA Transfection Kit (Stemgent), to either overexpress or inhibit human/mouse miR‐377‐3p. To this aim, mirVana^®^ miRNA mimic (#4464066/MC10524), mirVana^®^ miRNA inhibitor (#4464084/MH10524) of human/mouse miR‐377‐3p or the corresponding control oligos (#4464058), all from Thermo Fisher Scientific, were used. The transfection efficiency was evaluated using fluorescent RNA oligos (FAM3™ Dye‐Labeled Pre‐miR Negative Control, Thermo Fisher Scientific #AM17121) and a flow cytometer FACSCanto II (Becton, Dickinson and Company).

### 2.10 Assessment of the expression of PTPs in EPCs

Total RNA was extracted from cultured EPCs with High Pure PCR Template Preparation kit (Roche) and the level of RNA was determined with a Nanodrop ND‐1000 Spectrophotometer (Thermo Scientific). One microgram of total RNA was reverse transcribed using RT2 First Strand kit (SA Biosciences) and resulting cDNA was diluted 10 times before the real‐time PCRs. qPCR was performed using SensiFast SYBR Hi ROX kit (Bioline), specific primers and ABI 7900HT instrument. The values were normalized to the geometric mean of four house‐keeping genes (actin, calnexin, glyceraldehyde 3‐phosphate dehydrogenase and the proteasome subunit β type‐3). The comparative C_T_ method was used to quantify the results, assuming a 100% reaction efficiency.

### 2.11 Alteration of VE‐PTP/PTPRB expression level in EPCs

For VE‐PTP/PTPRB alteration in EPCs, cells were transfected with either pCDNA3.1 + /C‐(K)‐DYK vector (GenScript‐GenEZ ORF, clone Ohu21064D) containing VE‐PTP gene, or RNAi PTPRB (PRPRBHSS108847, Invitrogen), using Lipofectamine 3000 (Invitrogen/Thermo Fisher Scientific).

### 2.12 Statistical analysis

Data were analysed with GraphPad Prism 5.0 (GraphPad Software, Inc.) and presented as mean ± SEM. Comparison of multiple groups was made by ANOVA. Two‐group analysis was carried out by Student's *t* test. Probability values (*P*) <.05 were considered significant (*P < .05; **P < .01; ***P < .005).

## 3 RESULTS

### 3.1 Human EPCs engraft into *ex vivo* murine myocardial slices

Endothelial progenitor cells are normally grown in EGM‐2 complete medium,^11^ whereas cardiac cells are routinely cultured in complete DMEM, IMDM or F12 culture medium.^12^, ^13^, ^14^ The first issue before putting in direct contact two cell types that preferentially request different culture media was to choose the culture medium that provided support for survival and proliferation of both cell types *in vitro*. As DMEM complete medium has been proven unable to sustain EPC proliferation *in vitro* (Figure S1), comparative analysis of EGM‐2 and IMDM was performed to evaluate their capacity to sustain the viability of cardiac tissue slices in vitro.

To this aim, 300 μm‐thick fresh transversal slices of mouse heart were incubated in 24‐well plate (1 slice/well) in complete IMDM or EGM‐2 for indicated times (from 2 hours to 7 days) and then processed for MTT assay. The results showed that, in contrast to freshly prepared heart slices (analysed within 2 hours from the isolation), a substantial decrease in tissue viability was noted during the first 24 hours of incubation *in vitro* in the absence of oxygen bubbling (Figure S2). However, a certain extent of proliferation was still noted within the surviving cells during the next days of culture, irrespective of the culture medium used, demonstrating that the tissue remained viable in this hypoxic set‐up. These results suggested that *in vitro* culture of cardiac tissue slices in EGM‐2 mimicked the *in vivo* context of the hypoxic myocardial damage and might be further used as an appropriate system to evaluate the capacity of EPCs to improve the cardiac performance induced by ischaemic diseases.

**Figure 1:**
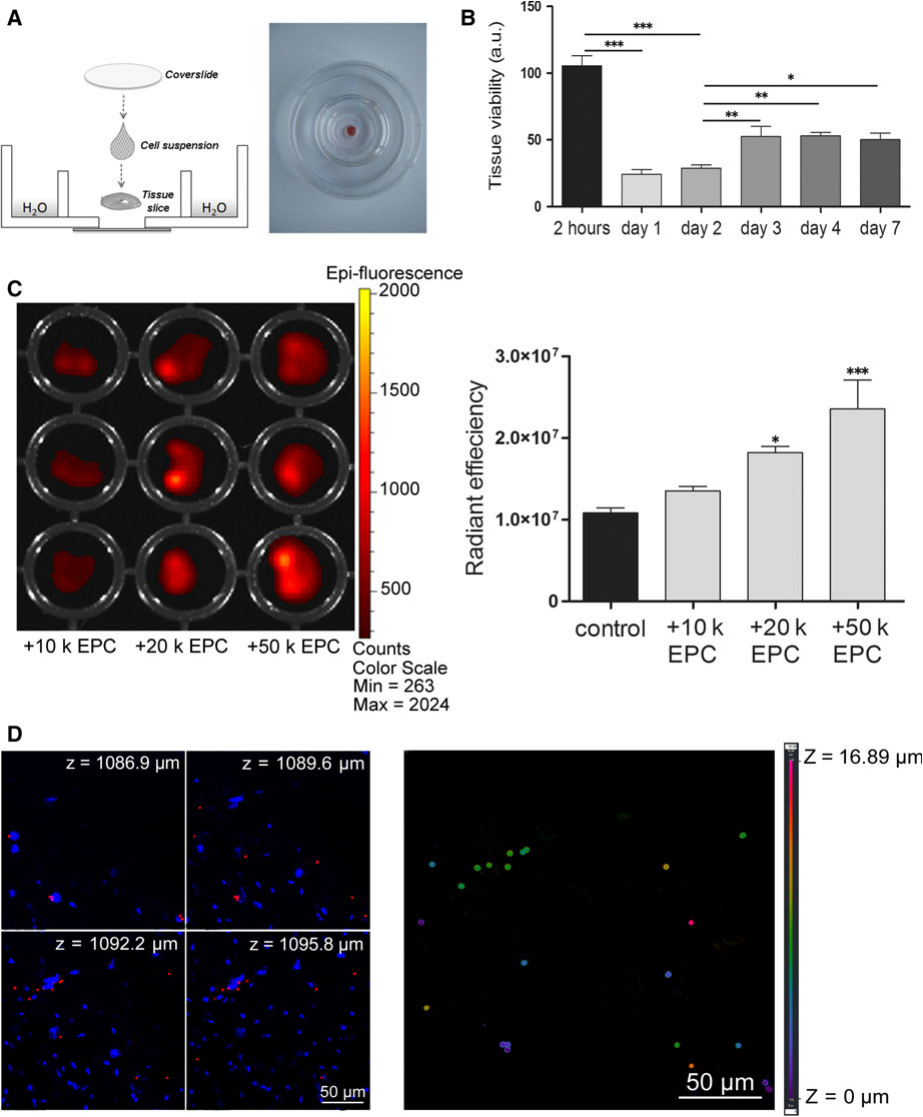
EPC engraftment into the myocardial slices. A, Schematic representation of the co‐culture system. B, Viability of cardiac slices in the co‐culture model *in vitro* (n ≥ 3). C, Tissue slices at 24 h after co‐culture with increasing number of Flash‐Red beads‐stained EPCs. Diagram at the right illustrates the quantification of cells adhered onto or into the myocardial slices (n ≥ 3). D, Confocal images showing the presence of Flash‐Red beads in different depths of the tissue (left image) and a colour‐based representation of the Flash‐Red beads localization on z‐axis (right image)

We next explored the *in vitro* conditions that allowed the engraftment of EPCs into the cardiac tissue in direct co‐culture. To this aim, EPCs were directly seeded onto cardiac slices in 96‐well plates, but this condition resulted in the adherence of cells onto the well surface rather than onto tissue slice, which remained floating (data not shown). Furthermore, xCELLigence studies using EPCs co‐cultured in direct (on E plates) or indirect (on CIM plates) contact with cardiac tissue indicated a rather inhibitory effect of the cardiac tissue on EPC adherence and proliferation (data not shown). This lack of affinity between adult cardiac tissue and EPC imposed us to create another co‐culture system that ensures an intimate contact between cells and tissue, in order to increase the probability of interaction between the two co‐culture components, while limiting the adherence of cells to the culture plate. To this aim, a co‐culture system was developed as described under Materials and Methods and illustrated in Figure 1A. The viability of tissue slices cultured in this system (Figure 1B) was maintained at similar levels as in the classical system with EGM‐2 (Figure S2), thus demonstrating the feasibility of this co‐culture model.

To quantify the engraftment of EPCs into the tissue, heart slices were co‐cultured with increasing number of Flash‐Red beads labelled EPCs (10.000‐50.000 cells per slice) followed by slice washing and evaluating for the presence of cells. IVIS analysis revealed the presence of EPCs onto tissue slices and showed that the fluorescent signal of slices increased in a dose‐dependent manner with the cell number seeded in co‐culture (Figure 1C). Further analysis of myocardial slices by confocal microscopy on z‐axis showed not only the adherence, but also the migration of EPCs into the depth of the myocardial slices (up to 17 μm distance downwards), thus demonstrating the engraftment of EPCs into the myocardial tissue in our co‐culture system (Figure 1D).

### 3.2 Co‐culture of EPCs with hypoxic heart slices confers protective paracrine effects on cardiac tissue *in vitro*

**Figure 2:**
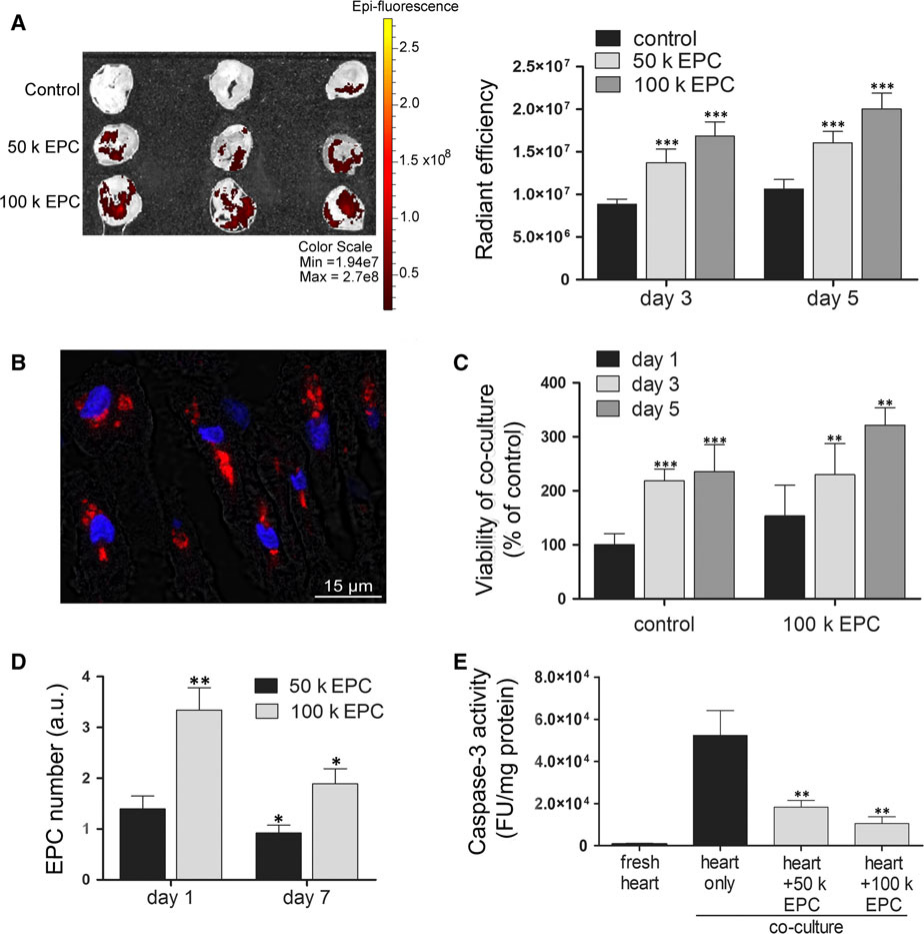
The fate of endothelial progenitor cells (EPCs) after interaction with cardiac slices *in vitro*. A, EPC engraftment into the ischemic myocardial tissue. Diagram at the right illustrates the quantification of the fluorescent signal at day 3 and 5 after 24‐h co‐culture of EPCs with cardiac slices (n ≥ 3). B, Representative image of a cardiac section illustrating the persistence of EPCs engrafted in the depth of ischemic adult myocardial tissue after 7 d of co‐culture. EPCs were labelled with CM‐Dil‐Vybrant in red and blue staining indicates Hoechst‐stained cell nuclei. C, The viability of co‐cultures using MTT assay (n ≥ 3). D, Quantitative PCR for human‐specific Alu sequences in mouse heart co‐cultured with EPCs (n = 3). E, Caspase‐3 activity in co‐cultures of adult myocardial tissue and EPCs at day 7 (n = 3)

To assess the viability and proliferation of EPCs after engraftment into the hypoxic heart slices, individual slices maintained for 24 hours in co‐culture with Vybrant‐CM‐DiI labelled EPCs were washed and further incubated in 96‐well plates for additional 3 or 5 days. IVIS analysis revealed the persistence of the fluorescent signal in the heart slices, thus certifying the cell survival after integration (Figure 2A). This data was further sustained by confocal analysis of heart slices, showing the presence of fluorescent cells inside the cardiac tissue (Figure 2B), which thus confirmed that the fluorescence was not leaked out in the tissue. It is worth mentioning that EPCs did not organize into capillary‐like structures, being rather distributed as single events dispersed between the resident cells of the cardiac slice.

To evaluate whether these cells proliferate and contribute to tissue regeneration after engraftment, MTT assay was performed on cardiac slices at different time‐points after 24‐hour co‐culture with EPCs. The results showed that the overall cellularity of the co‐cultures increased over time with the same magnitude in either the presence or absence of EPCs (Figure 2C). This data suggested that the proliferation was attributed to the host cardiac cells (most probably, the cardiac fibroblasts), rather than the donor cells. In corroboration with this data, quantification of human‐specific Alu sequences at 1 and 7 days after co‐culture initiation illustrated not only the absence of proliferation of grafted EPCs, but a decline in the amounts of grafted cells over time (Figure 2D). However, evaluation of the Caspase‐3 activity, an indicator of the apoptotic process, in the heart slices revealed a decreased apoptosis in the hypoxic heart slices after 7 days in culture in the presence of EPCs as compared to heart slices without EPCs (Figure 2E). This anti‐apoptotic effect was even amplified when a higher number of cells were used in co‐culture (ie, 65.12 ± 6.15% and 79.99 ± 0.57% less apoptosis of cardiac slices in the presence of 5x10^4^ EPC and 10^5^ EPC, respectively).

Together, these data indicated that co‐culture of cardiac slices with EPCs *in vitro* resulted in a likely transient engraftment of EPCs within the hypoxic host tissue, which did not proliferate after integration, yet produced a significant paracrine protective effect on the hypoxic cardiac slices.

### 3.3 Collagen deprivation stimulates expression of miR‐377 which impedes the angiogenic properties of EPCs

The above results indicated that conditions used for co‐culture of EPCs with hypoxic cardiac tissue provided EPCs a favourable environment to unveil the protective functions that previously entitled them as valuable cells for tissue regeneration. Keeping in mind the two important prerequisites for *in vitro* maintenance of EPCs (growth factors and collagen feeder layer), which have both been encountered in our co‐culture system, we assumed that myocardial scar was critical for EPC proper functioning *in vitro* and/or *in vivo*.

To get insights into the responsible factors and the mechanisms by which EPCs exerted their paracrine protective function on the hypoxic cardiac slices, the expression levels of angiogenesis‐related miRNAs were evaluated in EPCs, with emphasis on the particular effect of the collagen on EPC angiogenic properties.

**Figure 3:**
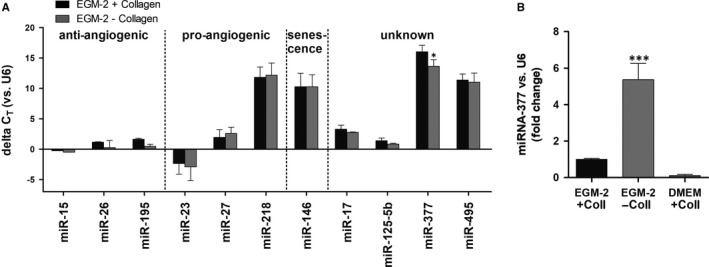
Expression of miRNAs in endothelial progenitor cells (EPCs) and anti‐angiogenic role of miR‐377. A, The relative expression level of several miRs in EPCs as determined by quantitative PCR. snRNA U6 was used for data normalization (n = 4). The miRNAs were grouped based on their role in angiogenesis: pro‐angiogenic, anti‐angiogenic, pro‐senescent or unknown. B, Quantification of miR‐377 in EPCs grown in culture in EGM‐2 complete medium, in the presence (EGM‐2+ Coll) and absence (EGM‐2− Coll) of collagen, or in complete DMEM in the presence of collagen (DMEM + Coll) (n = 3)

An inventory of angiogenesis‐related miRNAs has been established based on previous reports on miRNAs expressed significantly in endothelial cells (ECs): miR‐15, ‐17, ‐23, ‐26, ‐27, ‐125‐5b, ‐195, ‐218, ‐377 and ‐495.^15^ These miRNAs were further ranked into miRNAs for which the biologic role in ECs was known (pro‐angiogenic, anti‐angiogenic and pro‐senescent) and miRNAs for which the role in ECs was unknown (Figure 3A). A first hint provided by RT‐PCR analysis was that, similar to mature ECs, EPCs expressed high levels of miR‐15, ‐23, ‐26, ‐27 and ‐195, with have a recognized role in angiogenic properties of the cells, and much lower levels (around 1000 times less) of miR‐377, with yet undefined function in ECs (Figure 3A). Interestingly, the deprivation of collagen feeder layer from EPC culture resulted in a 5.37 ± 1.56‐fold increase in miR‐377 expression level, with no significant impact on the other miRNAs analysed (Figure 3A,B).

### 3.4 VE‐PTP/PTPRB is a miR‐377 target with important role in collagen‐dependent angiogenic properties of EPCs

Given the fact that protein phosphorylation and dephosphorylation are central events in many signal transduction pathways that regulate key cellular processes,^16^, ^17^, ^18^ we turned our attention towards the role of protein tyrosine phosphatases (PTPs) as mediators in blood vessel remodelling and angiogenesis.^19^, ^20^, ^21^

**Figure 4:**
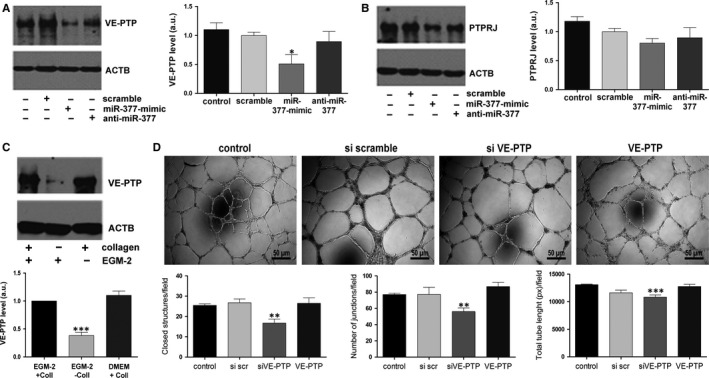
The role of VE‐PTP in endothelial progenitor cells (EPCs) and its regulation by miR‐377. A‐B, Western blot images and quantification of VE‐PTP(A) and PTPRJ (B) after miR‐377 alteration in EPCs. β‐Actin was used as loading control. C, Western blot images and quantification of VE‐PTP in EPCs grown in different culture conditions, that is in EGM‐2 complete medium, in the presence (EGM‐2+ Coll) and absence (EGM‐2− Coll) of collagen, or in complete DMEM in the presence of collagen (DMEM + Coll). ACTB is shown as loading control. D, The capacity of normal EPCs vs EPCs with altered levels of VE‐PTP to assemble into tube‐like structures in Matrigel *in vitro*.

First, the correlation between angiogenesis‐related miRNAs and PTPs was established based on theoretical predictions (Targetscan, miRwalk and Diana‐microT websites). Our analyses led to the identification with a high probability of VE‐PTP/PTPRB as a predicted target of miR‐377 (Figure S3). Reportedly, VE‐PTP is the only PTP specifically expressed in ECs.^19^, ^22^, ^23^, ^24^ Interestingly, we have also found it to be expressed in high amounts in EPCs, in comparison to other PTPs (Figure S4). The *in silico* prediction was confirmed in EPCs by gain‐ and loss‐of‐function assays. The efficiency of transfection with RNA oligonucleotides and the relative expression levels of miR‐377 after transfection with miRNA mimic or inhibitor are illustrated in Figure S5. To verify the specificity of VE‐PTP inhibition by miR‐377, PTPRJ, a PTP not regulated by miR‐377, but which was previously reported to be involved in endothelial differentiation and vasculogenesis^25^, ^26^ and for which post‐transcriptional regulation by miRNAs was also noted,^27^ was chosen as negative control. The results showed a 53.76 ± 14.50% decrease in the expression level of VE‐PTP, but not PTPRJ, in EPCs overexpressing miR‐377 mimic as compared to scramble control. As expected, no significant effect was noted in VE‐PTP (Figure 4A) and PTPRJ (Figure 4B) protein levels in EPCs treated with miR‐377 inhibitor.

In corroboration with the increase in miR‐377 expression level in EPCs grown *in vitro* in the absence of collagen, collagen deprivation also resulted in a substantial decay of VE‐PTP level (38.25 ± 5.82% of the control value, Figure 4C), thus supporting for the direct effect of miR‐377 onto VE‐PTP regulation.

Next, the effect of miR‐377‐regulated VE‐PTP on the angiogenic properties of EPCs was investigated by *in vitro* Matrigel assays. VE‐PTP silencing by small interfering RNA (siRNA) assay resulted in the deterioration of EPC angiogenic properties, as revealed by *in vitro* Matrigel assay (Figure 4D). Morphometric analysis showed that the number of tube‐like structures formed by EPCs in Matrigel decreased by ~25% (16.75 ± 4 vs 25.5 ± 2 closed structures/field, 56 ± 17.6 vs 77 ± 5 junctions/field and 10833 ± 769 vs 13063.6 ± 329 pixels total tube length formed by PTPRB‐silenced EPCs and control cells, respectively). These data indicated that the pro‐angiogenic properties of EPCs required the presence of collagen and pointed to miR‐377 as having an anti‐angiogenic role in these cells by negative regulation of VE‐PTP.

### 3.5 miR‐377‐VE‐PTP axis is involved in the cardioprotective effects conferred by EPCs in co‐culture system

**Figure 5:**
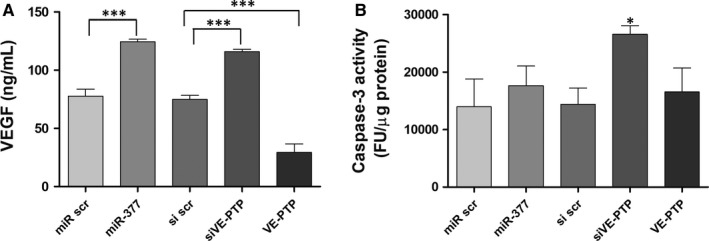
The protective function conferred by endothelial progenitor cells (EPCs) on the ischemic cardiac slices after alterations of miR‐377 and VE‐PTP. A, Murine VEGF quantification after 24 h of co‐culture between EPCs and cardiac slices. B, Caspase‐3 activity determined after 24 h of co‐culture between EPCs and cardiac slices

We next assessed the impact of miR‐377‐VE‐PTP axis in the protection conferred by EPCs on the hypoxic myocardium *in vitro*. To this aim, EPCs were modified *in vitro* by gain‐ and loss‐of‐function assays to modulate miR‐377 or VE‐PTP level, before being co‐cultured with mouse myocardial slices. The effect of modified EPCs on the hypoxic cardiac tissue was first evaluated by quantifying the secretion of mouse VEGF by the myocardial slices at 24 hours after co‐culture initiation. The results showed increased VEGF secretion by the myocardial tissue when co‐cultured in the presence of miR‐377‐overexpressing or VE‐PTP‐down‐regulated EPCs (Figure 5A). Thus, the secretion of mouse VEGF in the presence of miR‐377‐overexpressing EPCs was increased with 60% in comparison to control cells (124.4 ± 2.2 ng/mL in co‐culture with miR‐377‐overexpressing EPCs vs 77.7 ± 6 ng/mL in co‐culture with scramble transfected cells). On contrary, the overexpression of VE‐PTP decreased the secretion of VEGF by the myocardial slices (29.3 ± 4.28 vs 74.92 ± 1.98 ng/mL in scrambled control). These data indicated a diminished cardioprotection conferred by EPCs with altered expression of miR‐377, as a result of direct inhibition of VE‐PTP. Moreover, the anti‐apoptotic properties of EPCs on the myocardial tissue were also affected by interfering in miR‐377‐VE‐PTP axis. Thus, as illustrated in Figure 5B, the co‐culture of cardiac slices and modified EPCs in which the expression of VE‐PTP was repressed resulted in 1.9‐fold increase in Caspase‐3 activity, in comparison to native cells (26561.7 ± 1494.2 FU/μg protein in siVE‐PTP vs 13952.5 ± 4825.1 FU/μg protein in scrambled control).

Overall, the data showed that EPCs transiently engrafted and yielded pro‐angiogenic and anti‐apoptotic effects in hypoxic cardiac tissue in an in vitro model of interaction between hypoxic cardiac slices with EPCs. The collagen was important in these protective effects by preserving VE‐PTP levels, which were otherwise diminished by miR‐377.

## 4 DISCUSSION

In this paper, we designed a co‐culture system that assured an intimate contact between EPCs and hypoxic myocardial slices, which may be valuable for predicting the effect of transplanted stem cells onto the host myocardial tissue. The newly designed co‐culture system has allowed us not only to evaluate the protective effects conferred by EPCs onto hypoxic myocardium, but also to explore some aspects of the underlying mechanisms.

Similar to the clinical scenario, EPCs engrafted to some extent into host tissue and only transiently survived, yet produced visible protective effects, in terms of angiogenesis stimulation and protection against apoptosis. The attachment and/or engraftment of cells were dose‐dependent, a result that sustained the hypothesis “more cells to transplant better the outcome.” However, the engrafted cells did not proliferate locally, indicating that the protective effects on the hypoxic myocardium were of paracrine manner, by preventing the loss of myocardial tissue by apoptosis.

Using this co‐culture system, we showed here that: (i) normal EPCs maintained a low level of miR‐377, which was highly up‐regulated after collagen withdrawal from EPC culture; (ii) miR‐377 exerted an anti‐angiogenic role in EPCs and one of its specific target was VE‐PTP; (iii) the angiogenic and anti‐apoptotic properties of EPCs relied on VE‐PTP, whose expression was reduced after collagen withdrawal, by a pathway involving miR‐377.

Using EPCs in cellular therapy for myocardial regeneration after infarct is an appealing approach, considering the importance of revascularization on the cardiac function in the damaged tissue.^28^ However, the scar formation, a physiologically occurring event after myocardial infarction, was perceived so far as having a detrimental impact on the exogenously delivered stem/progenitor cells^29^ which might affect not only the engraftment, but also the general paracrine protective effects of stem cells. In this paper, we revealed a good face of the scar, in that the collagen, which was the main component of the myocardial scar, helped keeping a low level of miR‐377 and a high level of its target, VE‐PTP, and thus contributed to the protective effects of EPCs. This protective effect occurred in absence of EPC proliferation, which was in accordance to previous studies showing an increased level of VE‐PTP in resting cells as being associated to an increased capacity of ECs to organize into tubular structures in the 3D cultures.^30^ The mechanisms mediating the effect of collagen on EPCs are not definitively resolved, however they might involve integrin‐mediated signalling. Integrins have been indicated as major determinants of EPC homing, invasion, differentiation and paracrine factor production.^31^ Furthermore, it has been shown that EPCs overexpressing integrin β_1_ stimulated angiogenesis in ischaemic mouse hindlimbs, in which extracellular matrix proteins such as collagen I and fibronectin were up‐regulated.^32^

First, we investigated the expression of various miRNAs in EPCs, which were previously reported as being expressed in ECs and involved in blood vessels formation. Based on experimental evidence and *in silico* analysis, we found a connexion between VE‐PTP, a protein tyrosine phosphatase known to be involved in vessel remodelling and angiogenesis,^33^ and miR‐377. We showed that in the absence of the collagen I substrate, miR‐377 was up‐regulated, which determined a decrease in the VE‐PTP protein level. Next, we questioned whether these modifications *in vitro* had an impact on the capacity of EPCs to form tube‐like structures on Matrigel support, an indicator of their pro‐angiogenic effect.^34^ Our results indicated that the down‐regulation of VE‐PTP had a detrimental effect on the EPCs ability to form cords on Matrigel.

We took advantage of the EPC‐myocardial slice co‐culture system and evaluated the role of miR‐377‐VE‐PTP axis onto the anti‐apoptotic effect of EPCs in ischaemia‐affected myocardium. Our data revealed that manipulation of EPCs that diminished VE‐PTP or up‐regulated miR‐377 expression resulted in a detrimental cardiac protection conferred by EPCs, supported by the increased activity of caspase‐3, the main mediator of the apoptotic process^35^ and an increased secretion level of mouse VEGF, a marker of cardiac tissue hypoxia.

By concluding, our data suggest that VE‐PTP is an important player in the cardioprotective effects of EPCs. VE‐PTP level is regulated by the anti‐angiogenic miR‐377, whose expression level is kept low by collagen I, thus attributing to collagen‐based scar an important role for a positive outcome in the therapy of myocardial infarction. These observations remain to be validated *in vivo*. Such *in vivo* data will also increase the relevance of our co‐culture system for *in vitro* testing of various modulating agents aiming to improve the outcome of stem cell therapy for myocardial infarction.

## CONFLICT OF INTEREST

The authors confirm that there is no conflict of interest.
