# Supplementary material for: Collagen regulates the ability of endothelial progenitor cells to protect hypoxic myocardium through a mechanism involving miR‐377/VE‐PTP axis
Source: J Cell Mol Med. 2018 Jul 25;22(10):4700–8. doi: 10.1111/jcmm.13712 (PMC6156385; doi:10.1111/jcmm.13712)

**Supplemental data**

***Supplemental figure 1.*** Contrast-phase microscopy illustrating EPC proliferation in various culture conditions. Cells were seeded at the same density (6x10^3^ cells/cm^2^) in EGM-2 or serum-supplemented DMEM, with or without rat tail-collagen type I feeder layer and let to grow in culture for 5 days. One can note the inability of DMEM to sustain cell proliferation.


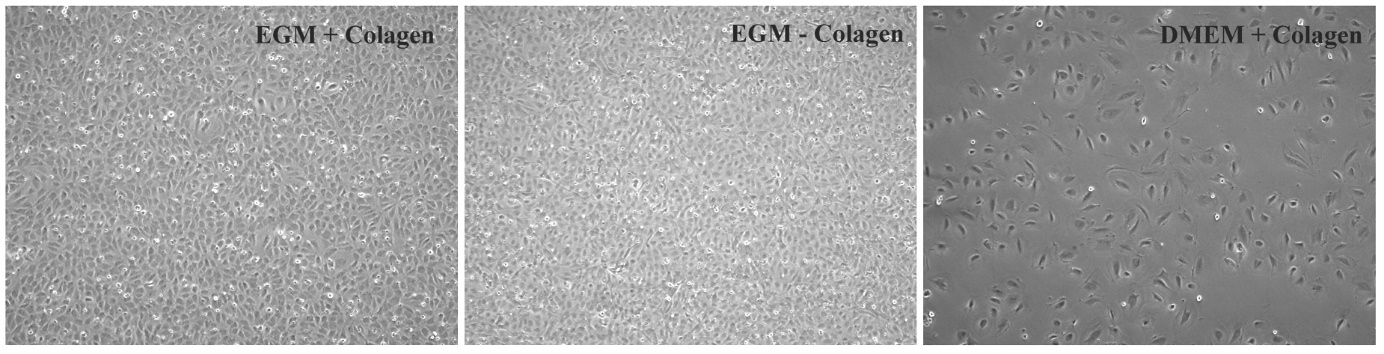


***Supplemental figure 2.*** Viability of murine myocardial slices in EGM-2 **A.** and IMDM medium **B.** using MTT assay, (n ≥ 3; one-way ANOVA).


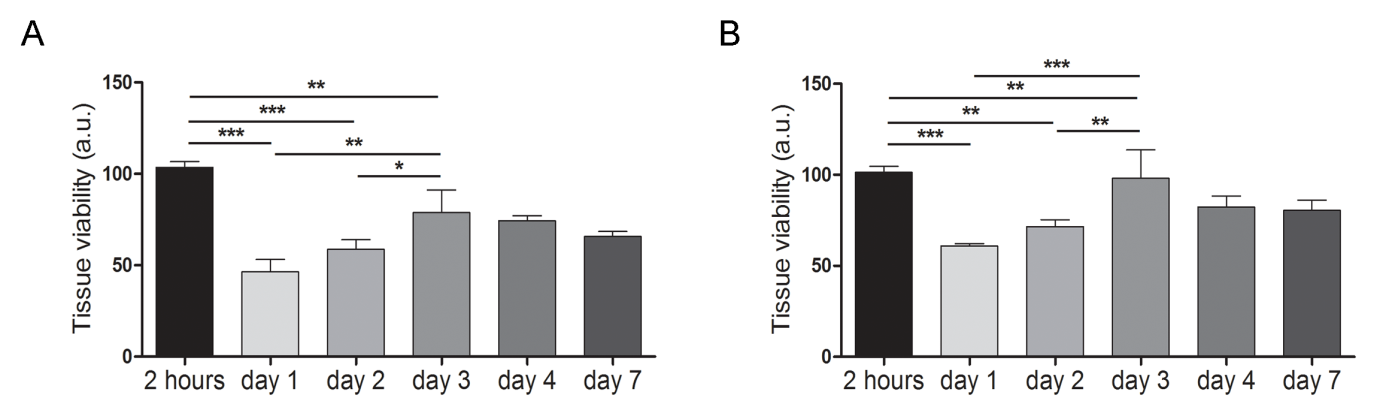


***Supplemental figure 3.*** Alignment of miR-377 with the 3’-UTR region of VE-PTP as predicted by targetscan.org. The seed region of miR-377 is underlined and is complementary with the sequence 39-42 of the VE-PTP 3’-UTR, highly conserved among mammals.

| Position 39-45 of VE-PTP 3' UTR  [hsa-miR-377-3p](http://www.mirbase.org/cgi-bin/mirna_entry.pl?acc=hsa-miR-377-3p) | **5' ...AUAAAAAUUAUUCACUGUGUGAU...**                      \|\|\|\|\|\|\|  **3'     UGUUUUCAACGGAAACACACUA** |
| --- | --- |

***Supplemental figure 4.*** Relative expression of PTPs in EPCs. Note the high expression level of VE-PTP gene in comparison to all others


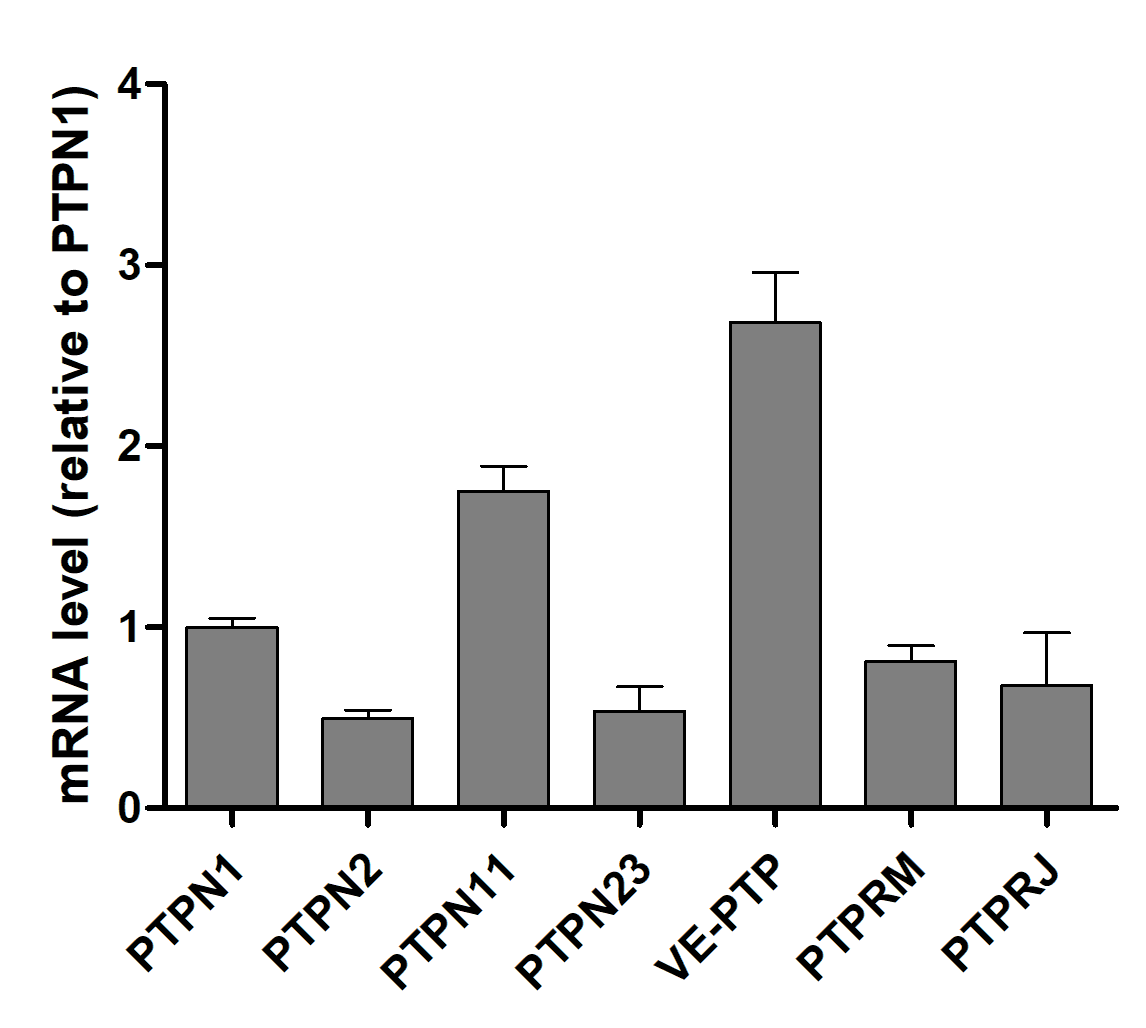


***Supplemental figure 5***. **A**. Representative dot plot images of EPCs transfected with FAM tagged RNA oligonucleotides (left) or without oligonucleotides (right), evaluated by flow cytometry. The transfection efficiency is shown as percentage of FAM positive cells. The non-viable cells were excluded by staining with 7-AAD (not showed) .**B.** Quantitative RT-PCR of miR-377 was performed from EPCs isolated 72 hours after transfection with the indicated oligonucleotides. Results are expressed as mean relative expression, 2-(Ct miR – Ct snoRNA202). MiR Control = Random sequence oligonucleotides.


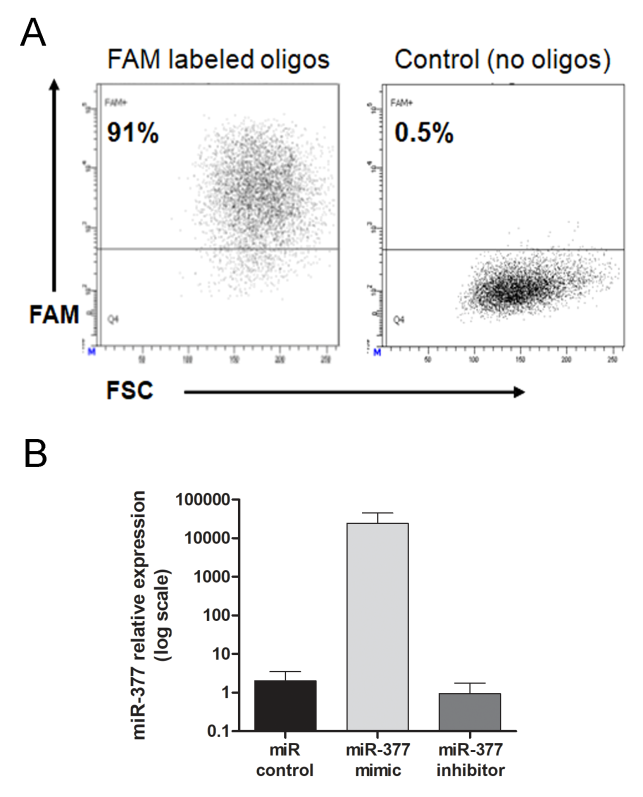

Supplement: Supplementary file 1 [file JCMM-22-4700-s001.docx]
